# Cardiopulmonary benefits of a cosmetic chest wall surgery

**DOI:** 10.1186/1749-8090-10-S1-A303

**Published:** 2015-12-16

**Authors:** Ghazi Elshafie, Babu Naidu

**Affiliations:** 1 Heart of England NHS trust, B95SS, Birmingham, UK

## Background/Introduction

There is contradictory evidence regarding improvement in dyspnea and exercise tolerance following corrective surgery for pectus carinatum. It is even more unclear as to the mechanism of any improvement.

## Aims/Objectives

We observed the changes in chest wall function in response to an incremental load exercise before and after surgery.

## Method

Using Optoelectronic Plethysmography, total and regional chest wall volumes were measured in 3 male patients with pectus carinatum who underwent a Ravitch procedure. Rib cage and abdominal volumes were recorded at rest and during exercise (incremental cycle ergometry), before and after surgery in conjunction with spirometry.

## Results

Our results shows that these patients end expiratory volume (EEV) increased by 14 +/- 0.4 % compared to quiet breathing during maximum exercise before surgery (P < 0.002). This dynamic hyperinflation or air trapping during exercise was corrected after surgery. Postoperatively their EEV decreased during maximum exercise by 2.3 +/- 1.4 % at 5 months respectively (P 0.001). The end inspiratory volume did not change significantly. This was associated with an 38 % increase in exercise time 5 months after surgical correction (P < 0.05).

## Discussion/Conclusion

This is the first published data to show dynamic hyperinflation in pectus carinatum patients and the beneficial effects of corrective surgery. This was associated with a significant improvement in exercise capacity after surgery. Therefore, we conclude that improvement in exercise capacity early after surgery is likely due to correction of dynamic hyperinflation. The longer term effects on chest wall function are yet to be defined.

